# Performance evaluation of the 3D-ring cadmium–zinc–telluride (CZT) StarGuide system according to the NEMA NU 1-2018 standard

**DOI:** 10.1186/s40658-024-00671-x

**Published:** 2024-07-25

**Authors:** Alessandra Zorz, Marco Andrea Rossato, Paolo Turco, Luca Maria Colombo Gomez, Andrea Bettinelli, Francesca De Monte, Marta Paiusco, Pietro Zucchetta, Diego Cecchin

**Affiliations:** 1 Medical Physics Department, Veneto Institute of Oncology IOV – IRCCS, Padua, Italy; 2 Unit of Nuclear Medicine, University Hospital of Padova, Padua, Italy; 3 Unit of Nuclear Medicine, Department of Medicine (DIMED), University Hospital of Padova, Padua, Italy

**Keywords:** SPECT/CT, CZT, Quality control, NEMA

## Abstract

**Background:**

The application of semi-conductor detectors such as cadmium–zinc–telluride (CZT) in nuclear medicine improves extrinsic energy resolution and count sensitivity due to the direct conversion of gamma photons into electric signals. A 3D-ring pixelated CZT system named StarGuide was recently developed and implemented by GE HealthCare for SPECT acquisition. The system consists of 12 detector columns with seven modules of 16 × 16 CZT pixelated crystals, each with an integrated parallel-hole tungsten collimator. The axial coverage is 27.5 cm. The detector thickness is 7.25 mm, which allows acquisitions in the energy range [40–279] keV. Since there is currently no performance characterization specific to 3D-ring CZT SPECT systems, the National Electrical Manufacturers Association (NEMA) NU 1-2018 clinical standard can be tailored to these cameras. The aim of this study was to evaluate the performance of the SPECT/CT StarGuide system according to the NEMA NU 1-2018 clinical standard specifically adapted to characterize the new 3D-ring CZT.

**Results:**

Due to the integrated collimator, the system geometry and the pixelated nature of the detector, some NEMA tests have been adapted to the features of the system. The extrinsic measured energy resolution was about 5–6% for the tested isotopes (^99m^Tc, ^123^I and ^57^Co); the maximum count rate was 760 kcps and the observed count rate at 20% loss was 917 kcps. The system spatial resolution in air extrapolated at 10 cm with ^99m^Tc was 7.2 mm, while the SPECT spatial resolutions with scatter were 4.2, 3.7 and 3.6 mm in a central, radial and tangential direction respectively. Single head sensitivity value for ^99m^Tc was 97 cps/MBq; with 12 detector columns, the system volumetric sensitivity reached 520 kcps MBq^−1^ cc^−1^.

**Conclusions:**

The performance tests of the StarGuide can be performed according to the NEMA NU 1-2018 standard with some adaptations. The system has shown promising results, particularly in terms of energy resolution, spatial resolution and volumetric sensitivity, potentially leading to higher quality clinical images.

**Supplementary Information:**

The online version contains supplementary material available at 10.1186/s40658-024-00671-x.

## Background

In nuclear medicine imaging, the introduction of cadmium-zinc-telluride (CZT) detectors is considered one of the most significant innovations of recent years [1, 2]. Semi-conductor detectors, such as CZT, convert gamma photons directly into electric signals, offering improved extrinsic energy resolution and count sensitivity as compared to conventional Anger systems which are based on thallium-doped sodium iodide NaI(Tl) detectors [3, 4]. Unfortunately, commercially available design with integrated collimator limits their viability for high-energy photon detection [5].

The first application of CZT detectors into clinical routine started with dedicated cardiac systems. Several articles have reported significant advantages in terms of both photon sensitivity and spatial resolution [4, 6], with considerable clinical improvements in terms of reduced acquisition time or administered activity [4, 7]. Patient dose optimization assumes relevant importance especially for oncological patients who undergo several examinations using ionizing radiation for tumor staging and follow-up.

More recently, 3D-ring general-purpose CZT SPECT/CT systems became commercially available, combining the advantages of pixelated semiconductor detectors with innovative geometries. Only two systems are currently available: the Veriton camera, the first 3D-ring CZT scanner, from Spectrum Dynamics (Caeserea, Israel) [7] and the StarGuide system, recently introduced by GE HealthCare (Haifa, Israel).

To enable standardized comparison among various γ-cameras, the National Electrical Manufacturers Association (NEMA) published the 'NEMA NU 1-2018 Standard for Performance Measurements of Gamma Cameras' [8]. At present, no specific standard for 3D-ring CZT SPECT systems are available. The NEMA NU 1-2018 standard could be employed to assess the performance of these systems. However, not all the tests are fully applicable due to systems’ ability to solely acquire 3D images, the presence of an integrated collimator and the pixelated nature of the detectors.

The aim of this study was to evaluate the performance of the StarGuide system, recently installed at the University Hospital of Padua (Padua, Italy), in accordance with the NEMA NU 1-2018 standard proposed with some adaptations tailored to the specific characteristics of this system [8]. Performance results will be compared to those reported in the literature for the SPECT/CT Anger system.

## Methods

**Fig. 1:**
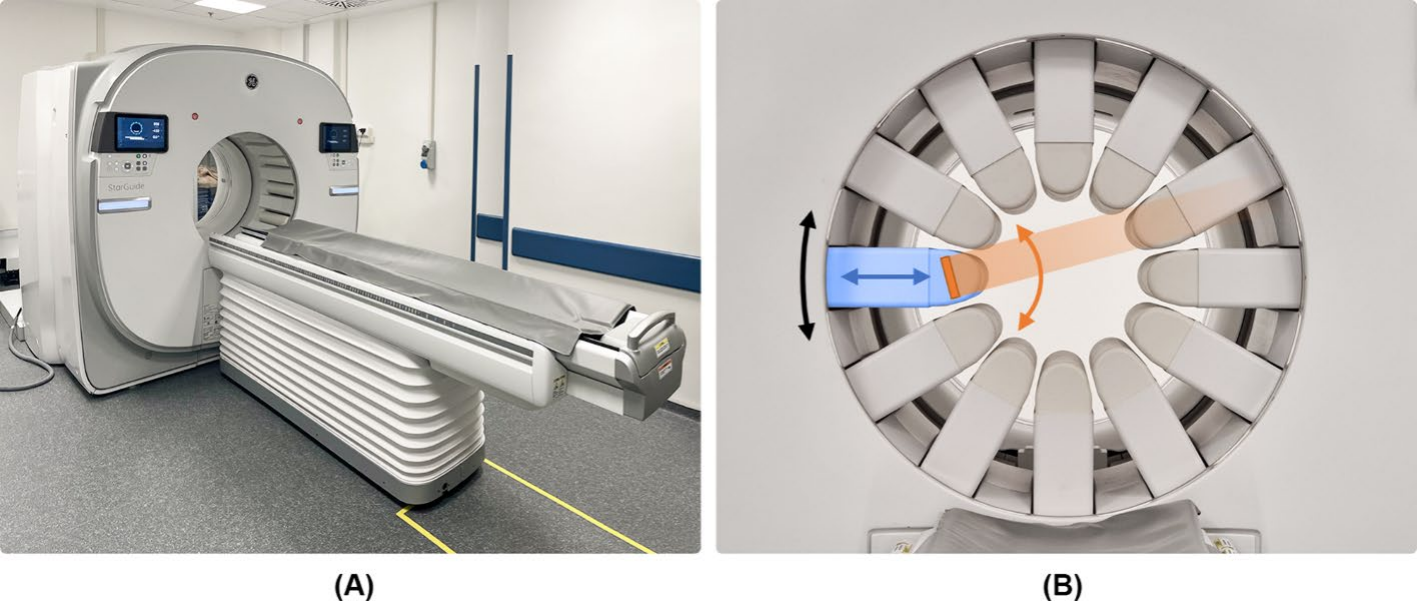
**A** The StarGuide camera and **B** its degrees of freedom: each column (in blue) can move with an individual radial motion (blue arrow) and with unison gantry circular motion (black arrow). Additionally, the detector (in orange) at the head of the column can move with sweep motion (orange arrow)

All tests were performed on the StarGuide SPECT/CT camera (GE HealthCare, Haifa, Israel) (Fig. 1A). The multi-detector system consist of 12 columns arranged in a 3D-ring configuration over an 80 cm diameter bore. Each column consists of seven modules of 16 × 16 CZT pixelated crystals with a dual-pitched integrated parallel-hole tungsten collimator. The collimator septa are aligned with each detector pixel.

The size of each of the 16 × 16 pixelated detector elements is 2.46 × 2.46 mm^2^, while the detector thickness is 7.25 mm.

Each detector column has different degrees of movements: an automated radial motion (in and out), a rotational motion along the gantry (from 2 to 6 steps for each bed position with a rotation range up to 25°) and a sweep motion (with a sweep range up to ± 15° in a step and shoot or continuous sweep mode) (Fig. 1B). Each detector moves independently from the others.

The longitudinal axial coverage of a single bed is 27.5 cm. Longer acquisitions are performed with multiple bed positions. The maximum whole-body SPECT scan range is equal to 195 cm plus additional 28 cm on the head support.

The energy range for the acquisition is [40–279] keV. The system operates in two energy ranges: the “low energy range”, for energies up to 200 keV, and a “medium energy” range, for imaging peaks above 200 keV. The two operational modes differ in the front end: in the low energy mode, each channel is equivalent to 0.5 keV, while in the medium energy mode, each channel is equivalent to 1 keV.

The system can acquire SPECT, gated SPECT, dynamic and whole-body scans with multiple SPECT fields of view (FOVs).

The SPECT acquisition consists of a combination of several steps of angular acquisition performed using gantry rotation. Within every step, each column radially moves towards the scanned body/object surface and each detector performs a sweeping motion to collect data from different projections. The acquisition mode can be set as ‘uniform’, where the detector sweep rate is constant over the FOV, or ‘focused’, where the detectors are forced to collect more data from a user-defined region of interest (ROI).

The system is equipped with a ring of optical sensors with an infrared transmitter and receiver diode array around the bore circumference at the front of the gantry. This allows the acquisition of an optical scout of the patient body contour every 0.5 cm.

The number of gantry rotation steps, the sweeping range and the detector radial position are automatically optimized by the system through to the acquisition of the optical scout. The height of the table is automatically adjusted to center the patient in the FOV.

Real time corrections for uniformity, energy, isotope decay and center of rotation are performed.

The system is equipped with two reconstruction algorithms: a standard Ordered Subset Expectation Maximization (OSEM) algorithm and Q.Clear, a Block Sequential Regularized Expectation Maximization (BSREM) algorithm [9] designed to enhance clinical image quality by simultaneously preserving edges while mitigating noise amplification. The size of the reconstructed pixels can be either 2.46 or 4.92 mm. During the post-reconstruction processing, StarGuide also supports a Clarity post-reconstruction filter (a noise-adaptive edge-preserving filter) with a contrast enhancement step. This option represents a sort of high-pass filter option that modulates the edge-preserving characteristics of the image when Clarity 3D is applied.

The system can acquire data in list mode, allowing retrospective reconstructions with reduced statistics or different energy windows.

The SPECT/CT system is integrated with the 16-row Optima 540 CT scanner.

Table 1 summarizes the features of StarGuide.

**Table 1:** StarGuide characteristics

Component	Characteristic	Specific
SPECT	Detectors	Pixelated CZT
	Columns (heads)	12
	N° modules/detectors per column	7
	N° pixels/modules	16 × 16
	Pixel dimension	2.46 × 2.46 mm
	Detector thickness	7.25 mm
	Detector axial coverage	27.5 cm
	Energy range	[40–279 keV]
	Collimator	Integrated tungsten parallel hole collimator
CT	N° rows	16

### NEMA NU 1-2018 tests

The NEMA NU 1 standard [8] used for performing SPECT performance tests was developed for conventional gamma camera systems based on Anger logic. The document acknowledges the emergence of novel SPECT systems which use discrete pixel detectors. Indeed, it incorporates some indications regarding the applicability of several tests procedures for pixelated and direct conversion detectors with fixed collimators.

Following these NEMA and the manufacturer recommendations, all the tests with the indications of their applicability or not to the StarGuide system are listed in Supplemental Table S1 of the supplemental materials. Due to the presence of an integrated collimator, all tests were performed extrinsically.

Prior to test acquisition, the detectors were calibrated in accordance with the manufacturer’s procedure, which corrects for the energy map and builds the uniformity maps. The center of rotation and SPECT-CT alignment were also verified.

All acquisitions were performed with the default energy window set by the manufacturer: for ^99m^Tc ± 10% centered on the photo-peak, for ^123^I + 6/− 5%.

Analysis were performed with a custom script provided by the manufacturer in MATLAB (MATLAB R2022b, Mathwork Natick, Massachusetts). For the tomographic contrast, an in-house developed script in MATLAB was used.

All the measures were repeated three times, except for the count rate performance in air which was performed only once. The average of the three measures and the range (minimum–maximum) were reported.

**Fig. 2:**
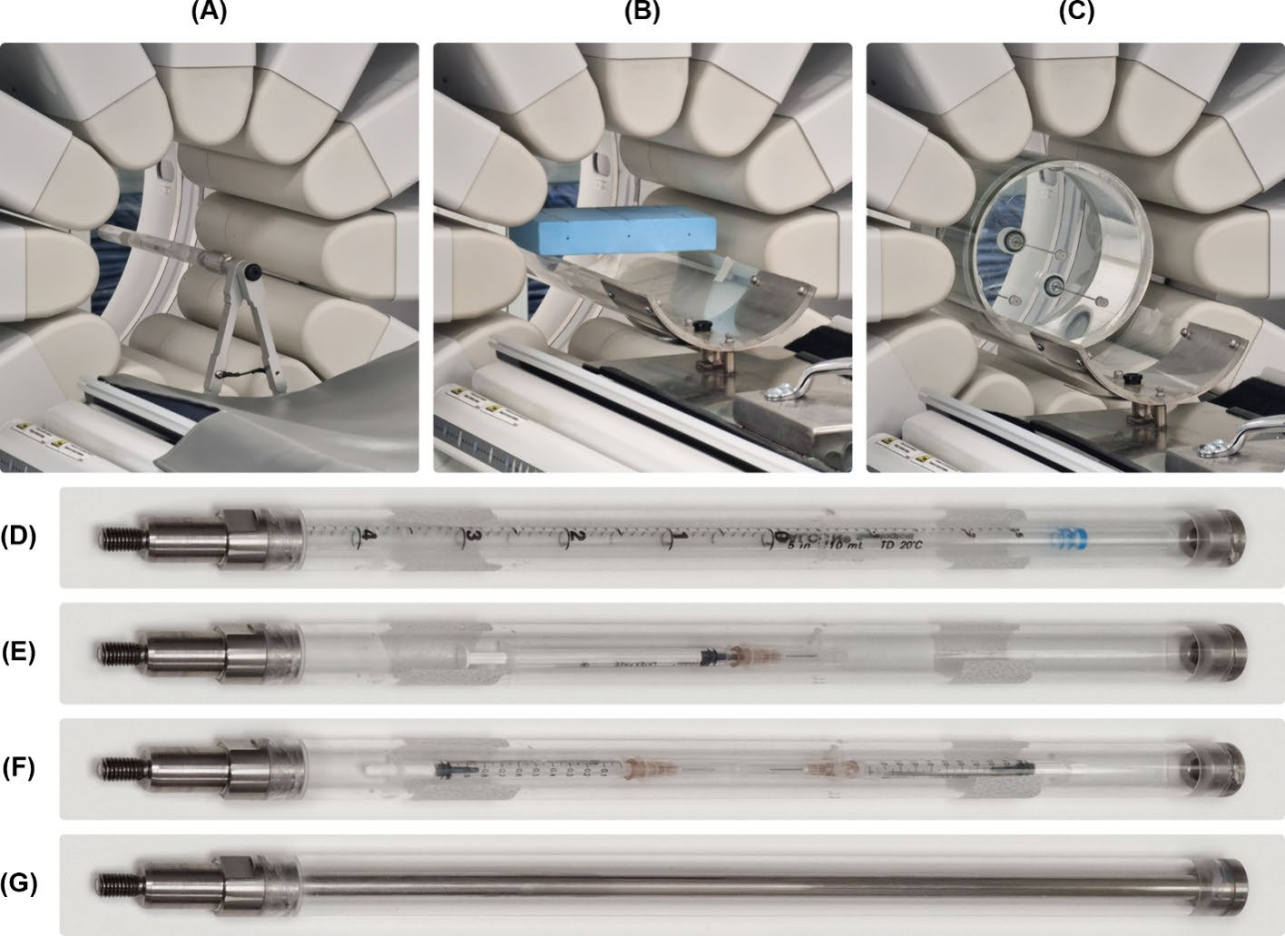
Experimental setup used for the following tests: **A** energy resolution, count rate performance in air, system spatial resolution without scatter and system planar sensitivity, **B** SPECT spatial resolution without scatter with a home-made support and **C** SPECT resolution with scatter. Different sources geometry used for the tests: **D** Line source in the axillary tube used for energy resolution, **E** single syringe in the axillary tube used for the count rate performance in air, **F** two 1-ml syringes in the axillary tube used for system planar sensitivity and **G** ^57^Co sealed linear source used for the flood field uniformity and the daily QC

*Energy resolution.* The measurement was performed extrinsically. The energy resolution was evaluated for ^57^Co, ^99m^Tc and ^123^I. Sources with linear geometry (Fig. 2D, G) were suspended in the center of the FOV with an axililary tube (Fig. 2A).

An acquisition without detector rotation and with sweep motion (range ± 15° and swipe duration of 10 s) of a total of 4.500 kcounts was performed for each detector column with different isotopes. The energy position of the peak was verified for each isotope. Energy resolution was expressed in terms of ratio of the photopeak full width at half maximum (FWHM) to the photopeak center energy, stated as a percentage, for each detector column. The FWHM was calculated in accordance with the NEMA NU 1-2018 instructions. The photopeak maximum was determined with a parabolic fit and the FWHM was obtained from the linearly interpolated half height channel values calculated at each side of the photopeak. The average of the FWHM measured for each detector was reported.

*Flood Field uniformity.* The measurement was performed extrinsically using the ^57^Co sealed linear source (Fig. 2G). The acquisition was performed without any detector rotation and with sweep motion (range  ± 15° and swipe duration of 10 s) for a total of 20.000 kcounts for each detector column. Adjacent detector pixels were summed to yield an effective pixel size of 4.92 × 9.84 mm^2^ (as suggested by the manufacturer), and subsequently, a 9-points convolution filter was applied. Differential and integral uniformities were calculated in the central and useful field of view (CFOV and UFOV) for each detector column. The highest values among the twelve measured values were reported. For pixelated detectors, the NEMA NU 1-2018 standard [8] recommends also reporting the number of defective pixels and the size of clustered defective pixels.

*Count rate performance in air.* The measurement was performed extrinsically using the decay source method. A syringe with approximately 800 MBq of ^99m^Tc was suspended in the center of the FOV with an axillary tube (Fig. 2A, E). The detector radius was set to 15 cm, the lowest achievable by the system. Repeated static acquisitions (i) were carried out. The start time ($${t}_{i}$$), the elapsed time of the measurements ($$\Delta {t}_{i}$$) and the number of counts ($${K}_{i}$$) were recorded for each measurement. The acquisition time of the first measurement was 10 s; the following measures were corrected for the decay of the source. Each data point was also corrected for the background count rate measured without the source ($${R}_{bkg})$$ with Eq. (1), obtaining the net counts $${C}_{i}$$. For each data point *i*, the observed count rate ($${OCR}_{i}$$) and the input count rate ($${ICR}_{i}$$) were calculated according to the Eqs. (2) and (3) following NEMA indications. Equation (2) corrects for the physical decay of the source during the measurements, while Eq. (3) extrapolates the count rate from time $${t}_{n}$$ where there is no time loss.

1 $$C_{i} = K_{i} - R_{bkg} \cdot \Delta t_{i}$$

2 $$OCR_{i} = \frac{{C_{i} }}{{\tau \cdot \left\{ {1 - \exp \left[ {\left( { - \Delta t_{i} } \right)/\tau } \right]} \right\}}}$$

3 $$ICR_{i} = OCR_{n} \cdot \exp \left\{ {\frac{{\left( {t_{n} - t_{i} } \right)}}{\tau }} \right\}$$

where $\tau$ is the mean lifetime given by $\tau = {\tau }_{half}/ln(2)$.

The values of the maximum OCR, the OCR at 20% loss and the OCR versus ICR curve were reported.

*System spatial resolution without scatter.* The measurement was performed with a line source of ^99m^Tc with approximately 150 MBq suspended in the center of the FOV with an axillary tube (Fig. 2A). The measurement was performed with the collimator radius set to 15 cm and the source in the center. The theoretical derivation of expected resolution at 10 cm ($R_{{100\;{\text{mm}},E}}$) from the collimator face (as requested by the NEMA standard [8]) was performed based on the Eq. (4) from “Physics in Nuclear Medicine”, 4th Edition, 2012 [10]:

4 $$R_{{100\;{\text{mm}},E}} = \frac{{100 + l_{eff,E} }}{{150 + l_{eff,E} }} \cdot R_{{150\;{\text{mm}},measured}}$$

where $R_{{150\;{\text{mm}},measured}}$ is the resolution measured at 15 cm, $l_{eff}$ is the effective collimator septal length which depends on the isotope measured (17.72 mm for ^99m^Tc).

A static acquisition of 10.000 kcounts without any detector rotation or sweep motion was acquired for each detector column. The system spatial resolution was calculated on the static image of each detector and expressed as the FWHM and FWTM of the line spread function for each detector column. The average of the twelve measured FWHMs and FWTMs were reported.

*System planar sensitivity.* Sensitivity was measured 15 cm from the collimator face using two 1-ml syringes with approximately 37 MBq of ^99m^Tc each positioned in the center of the FOV (Fig. 2A, F). The source activity inside the two syringes was accurately measured using a dose calibrator and summed to obtain ${A}_{cal}$ at the time of preparation (${T}_{cal}$). A static image of 40.000 kcounts was acquired for each detector column. The start time of the acquisition was recorded (${T}_{150}$). The decay-corrected total count rate for each detector was calculated in accordance with Eq. (5), where ${R}_{D=150}$ represents the decay-corrected count rate at 150 mm, ${C}_{D=150}$ the summed counts at 150 mm over the entire image, ${T}_{150}$ is the start time of the acquisition, ${T}_{cal}$ is the time of the activity calibration, ${T}_{acq,150}$ is the duration of the acquisition and $\tau = {T}_{half}/\text{ln}(2)$. The system planar sensitivity at 150 mm was calculated for each detector column in accordance with Eq. (6). The average of the twelve measured values was reported in cps/MBq.

5 $$R_{D = 150} = \frac{{C_{D = 150} }}{\tau } \cdot {\text{exp}}\left( {\frac{{T_{150} - T_{cal} }}{\tau }} \right) \cdot \left( {1 - {\text{ exp}}\left( { - \frac{{T_{acq,150} }}{\tau }} \right)} \right)^{ - 1}$$

6 $$S_{TOT} = \frac{{R_{D = 150} }}{{A_{cal} }}$$

*SPECT spatial resolution without scatter.* Three point sources of ^99m^Tc with a concentration of approximately 100 MBq/ml in glass capillary tubes with internal diameter smaller than 1 mm were used. The point sources were suspended in air using a dedicated extender and a home-made source-holder, with the last parallel to the tabletop (Fig. 2B). The central source was positioned on the axis of rotation and centered in the FOV. The other two point sources were 7.5 cm away from the central one point source, as required by the NEMA standard [8]. The acquisition was performed with a fixed radius of 16 cm that comprises two rotation steps, each of which acquires at a sweep range of ± 36°. The total acquisition time was 15 min. The images were reconstructed according to the “Nema Resolution” manufacturer reconstruction protocol with two different sets of parameters: OSEM algorithm, 10 iterations, 8 subsets, no filter, no correction for scatter or attenuation, a voxel size of 2.46 mm, clarity 3D post filter with power of 0.01 and Contrast Enhancement, and Q.Clear algorithm, 10 iterations, 8 subsets, regularization method based on Relative Differences for Maximum Prior (RDP) with gamma and beta value of 1 and 0.000001 respectively, no correction for scatter or attenuation, a voxel size of 2.46 mm, clarity 3D post filter with power of 0.01 and Contrast Enhancement. From the reconstructed volume, three orthogonal views were obtained by summing the volume data on the three sources following NEMA indications. The point spread function and the FWHM in X and Y direction for each of the nine point was calculated. The central transaxial, the central axial, the peripheral radial, peripheral tangential and peripheral axial average resolutions were calculated according to NEMA specifications.

*SPECT spatial resolution with scatter.* The NEMA triple line source phantom was used: a cylinder (inner diameter of 20 cm) filled with water with three line sources. The phantom was placed in the center of the system’s FOV using a special extender (Fig. 2C). Each line was filled with approximately 150 MBq of ^99m^Tc. Acquisition was performed without the use of the body contour with a fixed radius of 16 cm that included two rotation steps, each of which acquireds at a sweep range of ± 36°. The total acquisition time was 15 min. The images were reconstructed according to the “Nema Resolution” manufacturer reconstruction protocol with the same sets of parameters used for the SPECT spatial resolution without scatter. The FWHM resolution values of the central source and of the two peripheral sources were reported.

*System Volume sensitivity and Detector-detector Variations.* A 20-cm-diameter uniform cylindrical phantom filled with approximately 740 MBq of ^99m^Tc was placed in the center of the system’s FOV using a dedicated extender. Acquisition was performed without the use of the body contour with a fixed radius of 16 cm that included two rotation steps, each of which acquired at a sweep range of ± 36°. The total acquisition time was 15 min.

System volume sensitivity (SVS), average sensitivity per axial centimeter (VSAC) and maximum detector-detector sensitivity (DDS) variations were calculated from the twelve images in accordance with Eqs. (7), (8) and (9):

7 $$SVS = \frac{{A\left( {cts/sec} \right)}}{{B_{c} \left( {MBq/cc} \right)}}$$

8 $$VSAC = \frac{SVS}{{Length}}$$

9 $$DDS = 100 \times \frac{{c_{\max } - c_{\min } }}{{c_{\max } }}$$

where $A$ is the average counts per seconds of the SPECT acquisition obtained from image projections in the raw data, ${B}_{c}$ is the source activity concentration at the time halfway through the SPECT acquisition by applying the source decay correction factor for ^99m^Tc, $Length$ is the axial length of the cylindrical source, ${c}_{\text{max}}$ and ${c}_{\text{min}}$ are the maximum and the minimum total counts of each summed image from all the projections.

*Tomographic contrast.* The NEMA anthropomorphic thorax phantom (NEMA IEC Body Phantom Set, Data Spectrum Corporation) was used, which contains six fillable spheres (internal diameters of 13, 17, 22, 28, 28 and 37 mm). The four smallest spheres were filled with radioactive water, and the two largest with cold water, simulating both cold and hot lesions. A cylindrical insert filled with low density foam (density of 0.30 g/cm^3^) was fixed along the center of the phantom. A sphere-to-background-ratio (SBR) of 8–1 was achieved using a solution of approximately 200 MBq of ^99m^Tc and water in the NEMA IEC Body Phantom cavity volume to achieve an activity concentration of the background of 20 kBq/cc. SPECT images were acquired using two standard clinical protocols (10-min and 5-min FOV using the body contour). A CT scan was used for attenuation correction. The images were reconstructed according to the “Bone Torso” manufacturer reconstruction protocol used in clinical routine with Q.Clear algorithm, 10 iterations, 10 subsets, regularization method RDP with gamma and beta values of 2 and 1 respectively, scatter correction, voxel size of 2.46 mm, clarity 3D post filter with power of 0.01 and measured attenuation correction. A second reconstruction was also performed for both the 5- and 10-min acquisitions using the standard clinical protocol with OSEM algorithm, 2 iterations, 10 subsets, Butterworth post-filter with a cutoff frequency of 0.48 and a power of 10, scatter correction and measured attenuation correction. The in-house developed analysis software automatically places circular regions of interest (ROIs) with a diameter equal to the inner diameter of the spheres on each hot and cold sphere. ROIs of the same dimensions as those drawn on the hot and cold spheres were created in the background of the phantom on the slice centered on the spheres and on the slices as close as possible to ± 1 and ± 2 cm on either side of the central slice. The percentage hot and cold contrast recovery coefficient (${Q}_{H,j} \,\text{and}\, {Q}_{C,j}$), the background variability (${N}_{j}$) and the residual lung error (${\Delta C}_{lung,i}$) were calculated in accordance with Eqs. (10)–(15) following the NEMA NU-1 2018 standard [8].

10 $$Q_{H,j} = \frac{{\frac{{c_{H, j} }}{{c_{B,j} }} - 1}}{{\frac{{a_{H} }}{{a_{B} }} - 1}} \times 100\%$$

11 $$Q_{C,j} = \left[ {1 - \frac{{c_{c,j} }}{{c_{B,j} }}} \right] \times 100\%$$

where $c_{H, j}$ is the average counts in the hot ROI for sphere j, $c_{c,j}$ is the average counts in the cold ROI for sphere j, $c_{B,j}$ is the average background counts for all ROIs of size j, $a_{H}$, $a_{C}$ and $a_{B}$ are the activity concentrations in the hot sphere, in the cold sphere and in the background respectively.

12 $$N_{j} = \frac{{SD_{j} }}{{c_{B,j} }} \times 100\%$$

where ${SD}_{j}$ is the standard deviation of the background counts within ROIs (*K* = 60) equal to each sphere size j and ${c}_{B,j}$ is the average background counts for ROIs of size j.

13 $$SD_{j} = \sqrt {\mathop \sum \limits_{k = 1}^{K} \frac{{\left( {c_{B,j,k} - c_{B,j} } \right)^{2} }}{{\left( {K - 1} \right)}}}$$

14 $$c_{B,j} = \frac{1}{K} \mathop \sum \limits_{k = 1}^{K} c_{B,j,k}$$

15 $$\Delta C_{lung,i} = \frac{{C_{lung,i} }}{{C_{B, 37 mm} }} \times 100\%$$

where $C_{lung,i}$ is the average counts of the pixel value of each image *i* slice in a circular ROI of 30 mm diameter centered in the lung insert and $C_{{B, 37\;{\text{mm}}}}$ is the average of the sixty 37 mm background ROIs.

### Additional tests

A daily quality control (QC) test was performed prior to each NEMA test following the manufacturer indications. To verify the correct response of each detector, a ^57^Co sealed linear source (370 MBq at the calibration time) was positioned in the center of the FOV with a specific holder. The QC consists of low- and medium-energy tests, according to the two front end operational modes. Tests were carried out on the average and integral uniformity, the energy resolution, the peak position, the average good pixels and the maximum cluster size.

For image quality evaluation, a Jaszczak Phantom was also acquired according to AAPM TG 177 [11]. A Deluxe Flanged Jaszczak phantom filled with approximately 740 MBq of ^99m^Tc was positioned in the center of the FOV using a special extender. Acquisition was performed without the use of the body contour with a fixed radius of 16 cm that comprised two rotation steps, each of which acquired at a sweep range of ± 36°. The total acquisition time was 15 min.

The images were reconstructed according to the “Jaszczak” manufacturer reconstruction protocol with OSEM algorithm, 150 iterations, 2 subsets, no filter, voxel size of 2.46 mm and attenuation correction using the Chang method with a linear attenuation coefficient of 0.12/cm. The reconstructed phantom slices were visually inspected for spatial resolution, contrast and uniformity. For the spatial resolution, the composite image was obtained by summation of the 15 central slices where rods were visible. The smallest rod sector and the smallest sphere that could be visualized were identified. Images of uniform slices were inspected for specific artifacts.

## Results

### NEMA NU-1 2018 tests

*Energy resolution.* The measured energy resolution for ^57^Co, ^99m^Tc, and ^123^I are reported in Table 2.

**Table 2:** Energy resolution results for ^57^Co, ^99m^Tc, and ^123^I. FWHM: Full Width Half Maximum

Isotope	Average FWHM (Range)
^57^Co	6.02% (5.96–6.08%)
^99m^Tc	5.27% (5.27–5.28%)
^123^I	5.45% (4.75–6.48%)

*Flood Field uniformity.* The measured values for the differential and integral flood field uniformity are reported in Table 3 and represented in Supplemental Fig. 1. The NEMA standards suggest that, for pixelated detectors, the number of defective pixels and the size of clustered defective pixels should be reported. These values are reported in Table 8 as daily QC results.

**Table 3:** Results of the differential and integral flood field uniformity. The highest values from twelve detectors were reported

Parameter	Highest value (range)
Differential uniformity	0.70% (0.52–0.84%)
Integral uniformity	1.10% (0.95–1.26%)

**Fig. 3:**
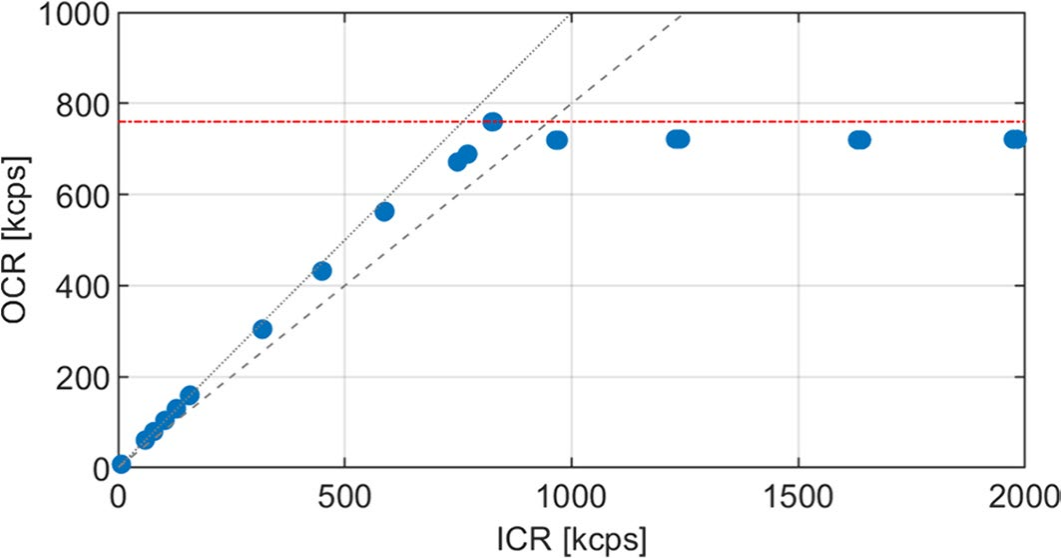
Results of the count rate performance in air. The observed count rate (OCR) are represented in function of the input count rate (ICR). The grey line represents the bisector and the dashed line the OCR = 0.8 × ICR line. The red horizontal line represents the maximum observed count rate

*Count rate performance in air.* Figure 3 shows the results of the count rate performance in air for the StarGuide system. The maximum count rate measured is 760 kcps and the ICR value at 20% loss is 917 kcps.

*System spatial resolution without scatter.* The measured system spatial resolutions in air at 15 cm and extrapolated at 10 cm distance for ^99m^Tc are reported in Table 4.

**Table 4:** Results of the system spatial resolutions without scatter at 10 cm and 15 cm for the ^99m^Tc line source

Average value at 10 cm (range)		Average value at 15 cm (range)	
FWHM	FWTM	FWHM	FWTM
6.96 mm	13.10 mm	9.91 mm	18.67 mm
(6.92–6.97 mm)	(13.09–13.11 mm)	(9.86–9.94 mm)	(18.66–18.69 mm)

*System planar sensitivity.* The average system planar sensitivity for ^99m^Tc is 97.1 cps/MBq (95.1–98.4 cps/MBq).

*SPECT spatial resolution without scatter.* The measured SPECT spatial resolution without scatter is reported in Table 5.

**Table 5:** SPECT spatial resolution (FWHM) without scatter for OSEM and Q.Clear reconstructions at central transaxial, central axial, peripheral radial, peripheral tangential, peripheral axial positions

Position	OSEM	Q.Clear
	Average value (range)	Average value (range)
Central transaxial	4.2 mm (4.0–4.5 mm)	4.3 mm (4.0–4.5 mm)
Central axial	3.5 mm (3.0–4.1 mm)	3.5 mm (3.0–4.1 mm)
Peripheral radial	3.4 mm (3.1–3.7 mm)	3.4 mm (3.1–3.7 mm)
Peripheral tangential	2.9 mm (2.8–3.0 mm)	2.9 mm (2.8–3.0 mm)
Peripheral axial	2.8 mm (2.8–2.9 mm)	2.8 mm (2.8–2.9 mm)

*SPECT spatial resolution with scatter.* The measured SPECT spatial resolution with scatter is reported in Table 6.

**Table 6:** SPECT spatial resolution (FWHM) with scatter for OSEM and Q.Clear reconstructions at central, radial and tangential positions

Position	OSEM	Q.Clear
	Average value (range)	Average value (range)
Central	4.1 mm (3.8–4.6 mm)	4.2 mm (3.9–4.6 mm)
Radial	3.6 mm (3.2–4.3 mm)	3.7 mm (3.2–4.2 mm)
Tangential	3.7 mm (2.9–4.3 mm)	3.6 mm (2.9–4.3 mm)

*System Volume Sensitivity and D-D Variations.* The measured SVS with ^99m^Tc is 524.5 kcps · MBq^−1·^ · cc^−1^ (520.4–528.6 kcps · MBq^−1^ · cc^−1^). The VSAC is 26.2 kcps · MBq^−1^ cm^−2^ (26.0–26.4 kcps · MBq^−1^ cm^−2^) and the maximum DDS variation is 23.3% (21.1–24.7%).

**Fig. 4:**
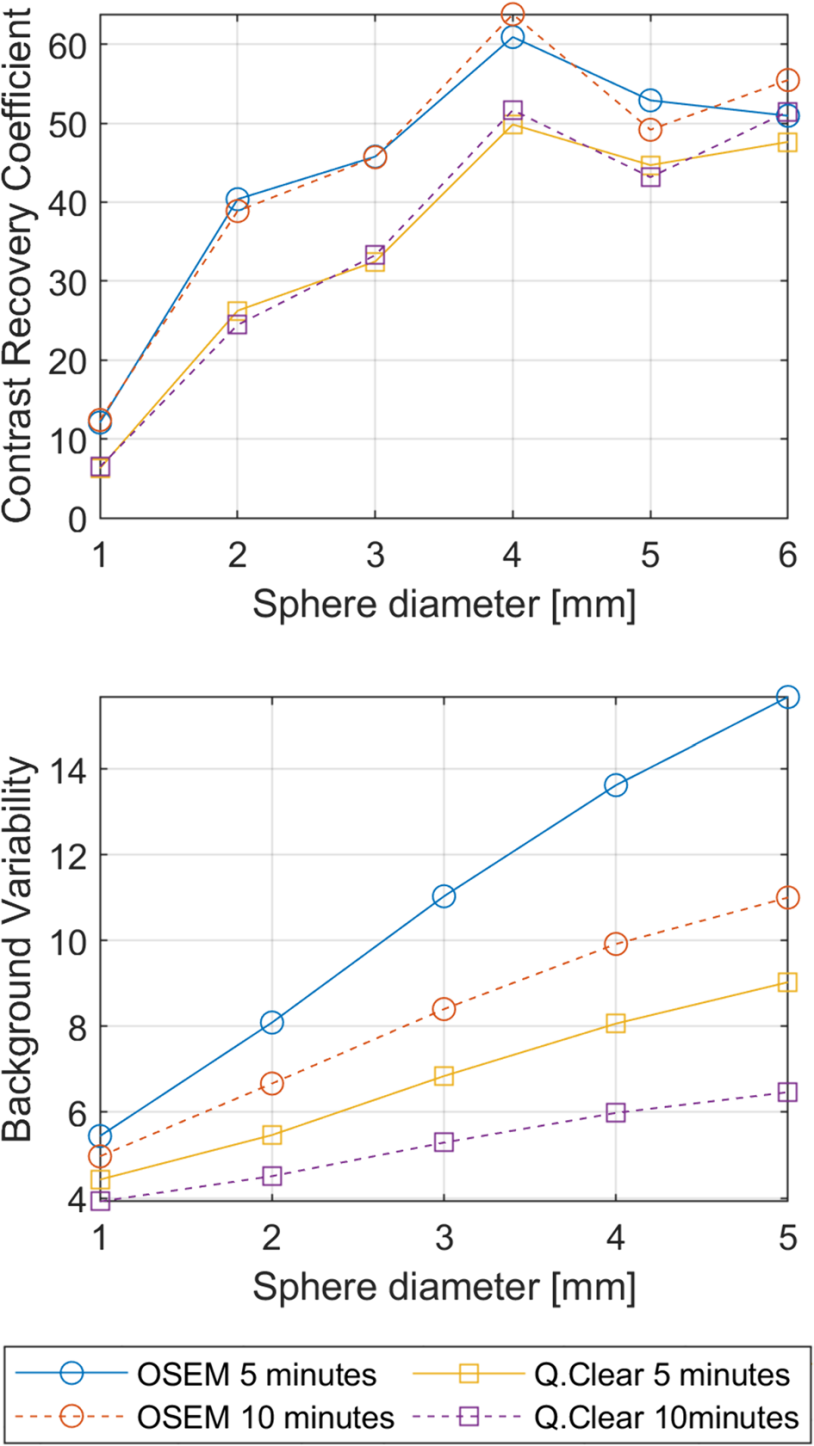
**A** Hot and cold contrast recovery coefficient for the OSEM and Q.Clear reconstructions of 5- and 10-min acquisitions and **B** Background Variability for the OSEM and Q.Clear reconstructions of 5- and 10-min acquisitions

**Fig. 5:**
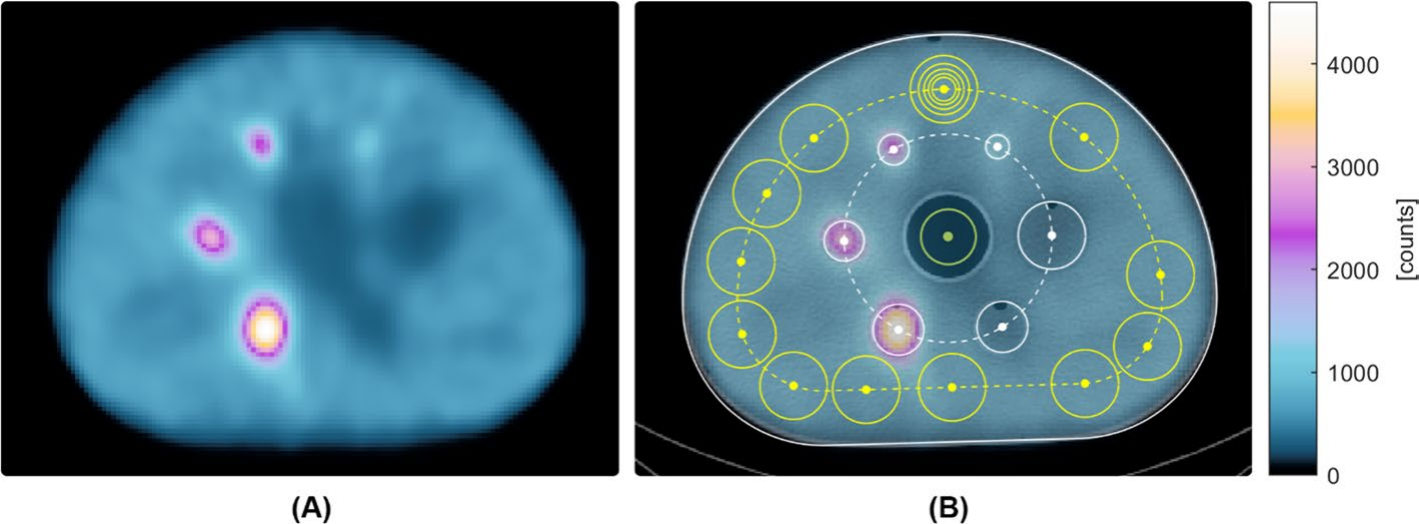
**A** Acquired SPECT image of the NEMA IEC body phantom and **B** the SPECT image superimposed on the CT scan. Sphere ROIs in white, background ROIs in yellow, lung insert ROI in green

*Tomographic contrast.* The measured hot and cold CRC, the BV and the lung error for OSEM and Q.Clear reconstructions for 5- and 1-min acquisitions are reported in Table 7 and Fig. 4A and B. Acquired images of the NEMA IEC phantom for the 10-min Q.Clear reconstruction with the ROIs used for the analysis are shown in Fig. 5. A comparison of the four different SPECT reconstructions is reported in Supplemental Fig. 2.

**Table 7:** Tomographic contrast results for OSEM and Q.Clear reconstructions of 5- and 10-min acquisitions. ${Q}_{H,j}$: hot contrast recovery coefficient, ${Q}_{C,j}$: cold contrast recovery coefficient, ${N}_{j}$: background variability, ${\Delta C}_{lung,i}$: residual lung error

Parameter	Sphere diameter (mm)	OSEM		Q.Clear	
		5-min FOV	10-min FOV	5-min FOV	10-min FOV
		Measured value (%)	Measured value (%)	Measured value (%)	Measured value (%)
${Q}_{H,j}$	13	12.1	12.4	6.3	6.5
	17	40.4	38.9	26.3	24.5
	22	45.8	45.7	32.5	33.3
	28	60.9	63.8	49.8	51.6
${Q}_{C,j}$	28	52.9	49.2	44.7	43.2
	37	50.9	55.5	47.6	51.5
${N}_{j}$	13	15.7	11.0	9.0	6.5
	17	13.6	9.9	8.1	6.0
	22	11.0	8.4	6.8	5.3
	28	8.1	6.7	5.5	4.5
	37	5.4	5.0	4.4	3.9
${\Delta C}_{lung,i}$		44.7	45.4	45.6	45.8

### Additional tests

*Daily QC.* The results of a daily QC are reported in Table 8. All daily QC tests performed during the performance tests resulted below the manufacturer-established tolerances.

**Table 8:** Daily QC results for low energy and medium energy operational modes

Energy	Parameter	Average value	Manufacturer tolerance
Low energy	Average differential uniformity (%)	1.0%	< 3.6%
	Average integral uniformity (%)	1.3%	< 4.5%
	Average energy resolution (%)	6.4%	< 7.5%
	Average peak position (keV)	121.9	120.6–123.6
	Average good pixels (%)	98.7%	93.8–100%
	Maximum cluster size	5	< 8
Medium energy	Average differential uniformity (%)	1.3%	< 3.6%
	Average integral uniformity (%)	2.4%	< 4.5%
	Average energy resolution (%)	7.2%	< 7.5%
	Average peak position (keV)	122.4	120.6–123.6
	Average good pixels (%)	98.7%	93.8–100%
	Maximum cluster size	5	< 8

**Fig. 6:**
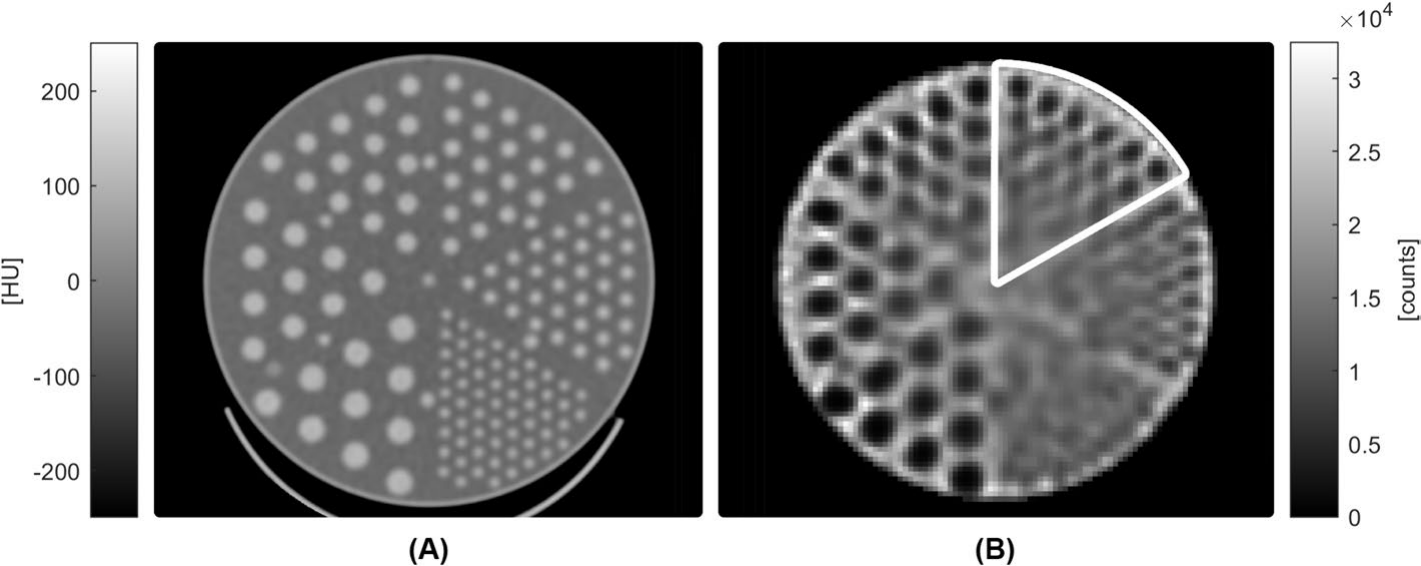
**A** CT and **B** SPECT images of the Jaszczak phantom with cold rods (diameters of 12.7, 11.1, 9.5, 7.9, 6.4, and 4.8 mm). In the SPECT image, rods are resolved down to the 7.9 mm diameter, encircled in white

*Image quality with Jaszczak Phantom.* Images of the cold rods of the Jaszczak phantom are shown in Fig. 6. Images of the cold spheres are shown in Supplemental Fig. 3. At least 12.7 mm sphere can be visualized, and 7.9 mm rods can be resolved. No artifacts were detected on uniform slices.

## Discussion

SPECT/CT has been a stable and established technique in nuclear medicine for many years [12–14]. The gamma camera principle of Hal Anger [15] remains the foundation of many installed SPECT devices; however, recently, a new 3D-ring CZT technology has been introduced. This innovation represents a paradigm shift in the clinical use of SPECT systems, providing only full 3D images.

The new ring geometry brings changes in QC management and performance evaluation. The performance evaluation tests recommended by the NEMA standard [8] are not fully applicable and must be adapted to its characteristics (as summarized in Supplemental Table S1 of the supplemental materials).

In particular, StarGuide is unable to acquire planar clinical images. All planar tests must be performed in a “quality control mode” and therefore not representative of the clinical situation. Furthermore, the system mounts an integrated collimator, which forces the tests to be performed extrinsically (e.g.: energy resolution, flood field uniformity, count rate performance). Some tests are not applicable due to the pixelated nature of the detectors, such as intrinsic spatial resolution, linearity, and multiple window spatial registration. Additionally, the minimal acquisition radius for the twelve detectors is 15 cm, which precludes the measurement of sensitivity and system spatial resolution at a 10 cm distance from the collimator surface. Whole body spatial resolution is also not applicable, as the system acquires a “step and shoot” mode. Finally, it should be noted that the system is not equipped with the Filtered Back Projection reconstruction algorithm. Consequently, all resolution test images were reconstructed with the iterative OSEM or Q.Clear algorithm.

In this study, we have summarized the performance of the 3D-ring CZT StarGuide system (GE Heatlhcare, Haifa, Israel) according to the recommendations of the NEMA NU 1 standard [8].

In terms of performance, one of the relevant results of this study is the measured energy resolution, which is around 5–6% for all the isotopes tested (Table 2). This is a twofold increase compared to the typical resolution of the NaI(Tl) gamma camera, which is around 10% [16, 17]. The increased energy resolution allows for a better definition of the photo-peak counts and consequently better scatter correction through the utilization of a narrow energy window for the acquisition. This improvement could be clinically useful for dual-isotope acquisitions. Some experiences have been already reported in literature with the CZT system [18, 19].

Flood field uniformity was assessed according to the NEMA standard [8], however the measurement was conducted extrinsically. Another discrepancy with regard to NEMA indication is the use of a rectangular pixel size instead of a square one for the analysis. The super-pixel size used in the test (4.92 × 9.84 mm^2^) is a manufacturer recommendation that should be followed in order to perform the analysis with the Matlab code and to verify the respect of the established tolerance in the acceptance test. Our results were below the manufacturer tolerances, established as 1.3% and 1.6% for differential and integral uniformity respectively. However, the pixelated nature of the detector must be taken into consideration in order to fully acknowledge the relevance of this QC: to ensure the overall system performance in terms of uniformity, it is crucial to evaluate the uniformity index linked with parameters related to each pixel’s status. Both the number of bad pixels and the maximum bad pixel cluster size are duly reported in the daily QC results. These parameters must be continuously monitored throughout the scanner’s life. According to manufacturer’s specifications, the number of bad pixels should remain below 6% and the maximum cluster size should be 8 pixels.

In terms of count rate response, there is a noticeable variation as compared to the Anger camera. StarGuide highlights the typical characteristics of a digital system: when the maximum count rate is reached at 760 kcps, the detector response remains constant (Fig. 3). Based on the maximum count rate measured for a source in air with all detectors closed at the minimum radius, it seems unlikely that clinical situations would involve dead time issues, even in the case of post-metabolic radiotherapy acquisitions.

One of the most remarkable results of this study is the SPECT spatial resolution and volumetric sensitivity. While the system spatial resolution (Table 4) is comparable to that of an Anger camera [16, 20], the measured SPECT resolution with and without scatter (reported in Tables 5 and 6) was approximately 4 mm in all directions, with superior values to those of a conventional system [16, 17]. This was also evident qualitatively in the Jaszczak phantom image (Fig. 6). There were no observed differences between Q.Clear and OSEM results for SPECT spatial resolution with and without scatter.

The system planar sensitivity (97 cps/MBq) is comparable to that of conventional gamma camera [16, 17], although the detector dimension is smaller. The measured volumetric sensitivities (520 kcps · MBq^−1^·cc^−1^) are higher than those achievable with the Anger camera [7]. This increase in sensitivity may result in lower acquisition time or patient administered activity [19, 21], as already demonstrated with dedicated cardiac CZT-based systems [22]. A reduction in the administered activity, and consequently in patient dose, is particularly beneficial for pediatric and oncological patients who are exposed to several radiological examinations during their lifetime or cancer care pathway.

NEMA 2018 [8] introduced tomographic contrast as a new QC measure for SPECT systems, which assesses cold and hot contrast in a warm background. Figure 5 and Supplemental Fig. 2 show images of the NEMA IEC phantom acquired using the StarGuide system, demonstrating promising contrast even for small lesions. A comparison of the 10- and the 5-min acquisitions indicates that the Q.Clear algorithm maintains consistent image quality, with minimal noise and contrast variations. This suggests the potential for exploring low dose or fast clinical protocols [19, 21]. Conversely, an increase in detector noise was more pronounced for OSEM reconstruction when reducing statistical counts. The recovery coefficients of the OSEM reconstruction appeared to be higher than those of the Q.Clear one, at the cost of an increased image noise, particularly evident in the 5-min acquisition. The ${Q}_{H,j}$ results were found to be superior to those obtained by Thibault [20] for the biggest sphere (41% for Discovery NM670 by GE Healthcare with LEHR collimator).

Overall, the tests showed several improvements in performance for the StarGuide compared to an Anger system. These enhancements offer potential clinical benefits such as shorter acquisition time and reduced injected activity and patient dose, as well as improved quantification accuracy for personalized dosimetry in radionuclide therapy for oncological patients [23]. Ideally, this type of scanner could also be eligible for standardization of absolute SPECT/CT quantification initiatives such as EARL accreditation for PET/CT [24], that has already been initiated with conventional SPECT/CT systems [25].

To the best of our knowledge, this is the first study that provides a comprehensive NEMA NU 1-based performance report of the 3D-ring CZT StarGuide system, describing which tests can be performed or not due to the specificity of this system (integrated collimator, ring geometry and pixelated nature of the detector). Only two other congress abstracts by Le Rouzic [26] and Ferri et al. [27] have been published previously.

Additionally, we only found two original articles by Desmonts et al. [7] and Bordonne et al. [28] that present a comparison between CZT systems and Anger cameras. Desmonts [7] and colleagues conducted phantom tests to compare the performance of the 3D-ring Veriton system (Spectrum Dynamics, Caesarea, Israel) and an Anger camera. The results showed that the CZT camera’s photon sensitivity was between 1.6 and 8 times higher than that of the Anger camera. The measured extrinsic spatial resolution was about 3.6 mm, comparable to the value obtained in this study. Similar results were also obtained in terms of energy resolution (5.33% for ^123^I and 5.46% for ^99m^Tc) and for Jaszczak phantom acquisition. Bordonne et al. [28] obtained similar results for CZT systems. The study revealed that the photon sensitivity of a Veriton camera was twice that of a conventional gamma camera for both phantom and patient studies, resulting in improved image contrast.

One limitation of this study is the lack of the optimization parameters of the reconstruction process. The implementation of the BSREM reconstruction algorithm, which has shown encouraging results in PET applications [29], is a novelty for SPECT images. The results of this study indicate that Q.Clear has no significant impact on SPECT resolution and image contrast. However, it does appear to have a notable effect on image noise, particularity in the case of low statistical count images. Future studies should focus on improving the performance of the BSREM algorithm, by fine-tuning of the reconstruction parameters to improve image contrast and further reduce noise.

## Conclusions

The introduction of 3D-ring CZT systems has significantly changed the scenario of single photon emission tomography imaging. The NEMA NU 1-2018 standard can be tailored to the characteristics of this novel system, such as the presence of a pixelated detector, a 3D-only acquisition geometry and the presence of an integrated collimator. The performance tests of the StarGuide system have shown promising results, particularly in terms of energy resolution, spatial resolution and volumetric sensitivity, improvements that could potentially lead to higher quality clinical images.

### Supplementary Information

Supplementary Material .

## Data Availability

The datasets generated and/or analyzed during this study will be available at the following 10.5281/zenodo.12759709.

## Abbreviations

BSERM: Block sequential regularized expectation maximization
BV: Background variability
CFOV: Central field of view
CRC: Contrast recovery coefficient
CT: Computed tomography
CZT: Cadmium-zinc-telluride
DDS: Detector–detector sensitivity
FBP: Filtered back projection
FDG: Fluorodeoxyglucose
FOV: Field of view
FWHM: Full width at half maximum
ICR: Input count rate
NaI(Tl): Sodium Iodide doped with Tallium
OCR: Observed count rate
OSEM: Ordered subset expectation maximization
PMT: Photomultiplier tubes
QC: Quality control
RDP: Relative differences for maximum prior
ROI: Region of interest
SPECT: Single photon emission computed tomography
SUV: Standardized uptake value
SVS: System volume sensitivity
UFOV: Useful field of view
VSAC: Volume sensitivity per axial centimeter
