# Supplementary material for: Performance evaluation of the 3D-ring cadmium–zinc–telluride (CZT) StarGuide system according to the NEMA NU 1-2018 standard
Source: EJNMMI Phys. 2024 Jul 25;11:69. doi: 10.1186/s40658-024-00671-x (PMC11272762; doi:10.1186/s40658-024-00671-x)

**Supplementary material**

**Supplemental Table 1.** List of NEMA tests [NEMA NU-1 2018] with the indication of their applicability to the StarGuide system.

| Section | Test | Conformance | Details |
| --- | --- | --- | --- |
| 2.1 | Intrinsic spatial Resolution | N.A. | Not applicable to pixelated detector |
| 2.2 | Intrinsic spatial Linearity | N.A. | Not applicable to pixelated detector |
| 2.3 | Intrinsic energy Resolution | Yes | Measurement performed extrinsically |
| 2.4 | Intrinsic flood field Uniformity | Yes | Measurement performed extrinsically |
| 2.5 | Multiple window spatial registration | N.A. | Not applicable to pixelated detector |
| 2.6 | Intrinsic count rate performance in air | Yes | Measurement performed extrinsically |
| 2.7 | Intrinsic spatial resolution at 75,000 CPS | N.A. | Not applicable to pixelated detector |
| 2.8 | Intrinsic flood field uniformity at 75.000 CPS | N.A. | Not applicable to pixelated detector |
| 3.1 | System spatial resolution without scatter | Yes | For practicality, the measurement is performed and analyzed per NEMA at 15 cm (not 10 cm) from collimator face. The minimal acquisition radius for the 12 detectors simultaneously is 14.3 cm |
| 3.2 | System spatial resolution with scatter | Yes | Performed on representative detectors using Static Acquisition |
| 3.3 | System spatial sensitivity and detector penetration | Yes | Sensitivity was measured at 15 cm from collimator face (not 10 cm) |
| 3.4 | Detector shielding | Yes |  |
| 3.5 | System count rate with scatter | Yes |  |
| 4.1 | System alignment | N.A. | Not applicable to non-planar geometry |
| 4.2 | SPECT reconstructed spatial resolution without scatter | Yes | Acquisition and reconstruction deviate from  NEMA instructions |
| 4.3 | SPECT reconstructed spatial resolution with scatter | Yes | Acquisition and reconstruction deviate from  NEMA instructions |
| 4.4 | System volume sensitivity | Yes | Acquisition deviates from NEMA instructions |
| 4.5 | Detector-Detector Variation | N.A. | Not applicable due to limited gantry rotation angles |
| 5.1 | Whole body spatial resolution | N.A. | Not applicable to non-planar geometry |
| 6 | Tomographic contrast  and Absolute quantification accuracy | Yes |  |
| 7 | SPECT/CT Co-registration accuracy | Yes |  |
| N.A.: Not Applicable. | | | |

**Supplemental Figure 1.** Integral flood field uniformity images of the twelve pixelated detectors.


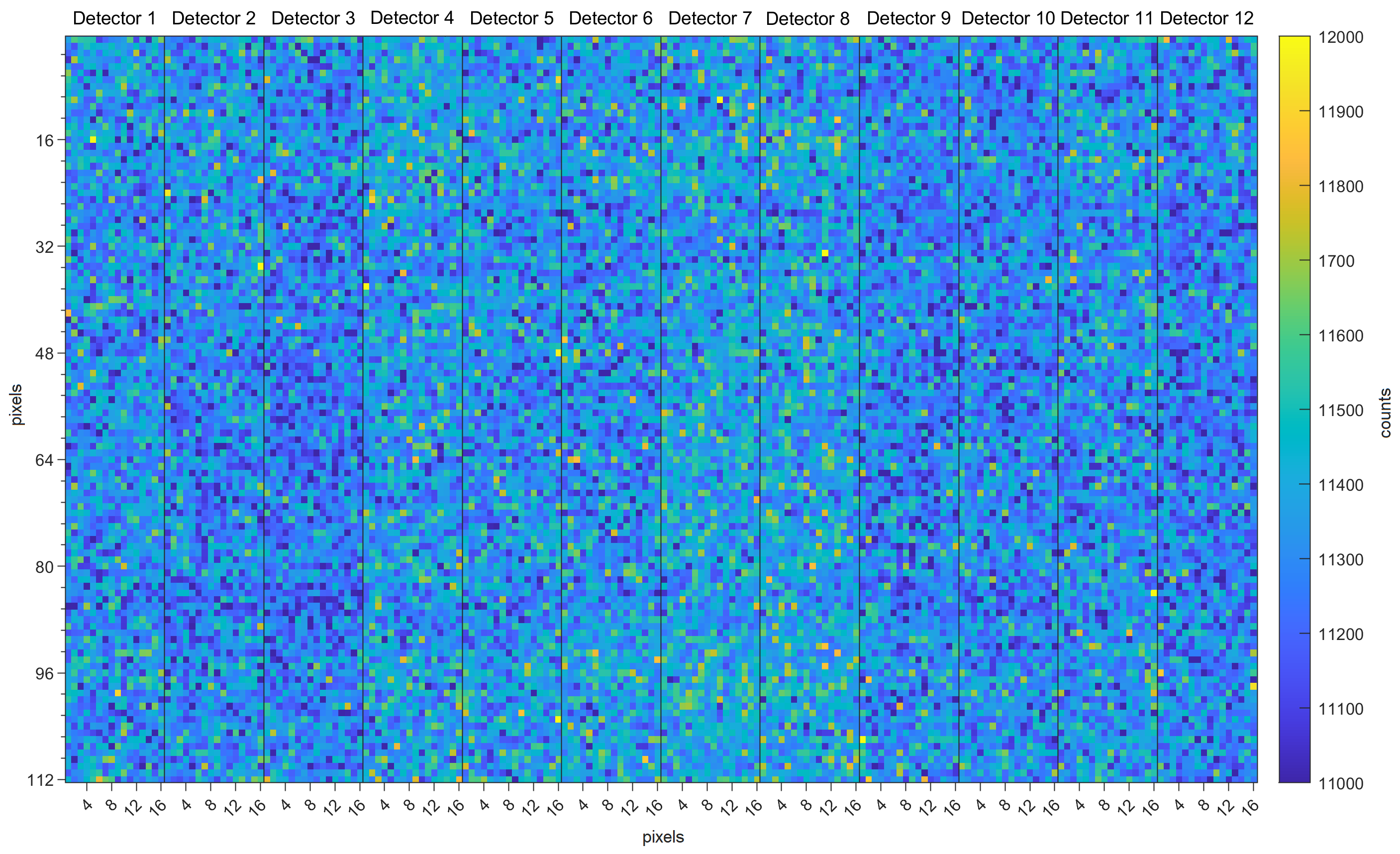


**Supplemental Figure 2.** Comparison of SPECT slices of the four reconstructions of the NEMA IEC Body Phantom Set: 5-minute and 10-minute acquisitions for OSEM and Q.Clear algorithm.


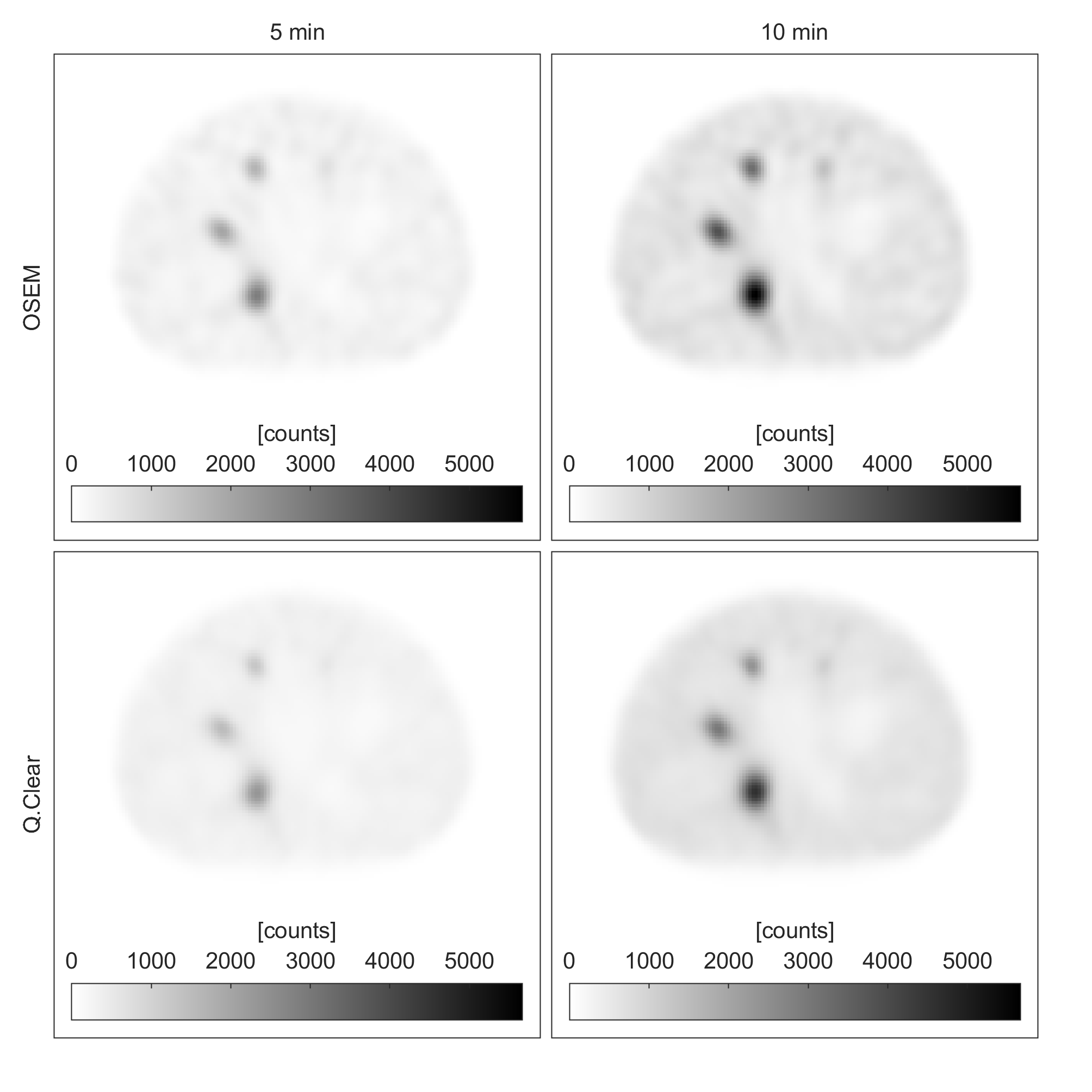


Supplemental Figure 3. (A) CT and (B) SPECT images of the Jaszczak phantom with cold spheres (diameters of 31.8, 25.4, 19.1, 15.9, 12.7, and 9.5 mm). The sphere with a 12.7 mm diameter is visible in the SPECT image.


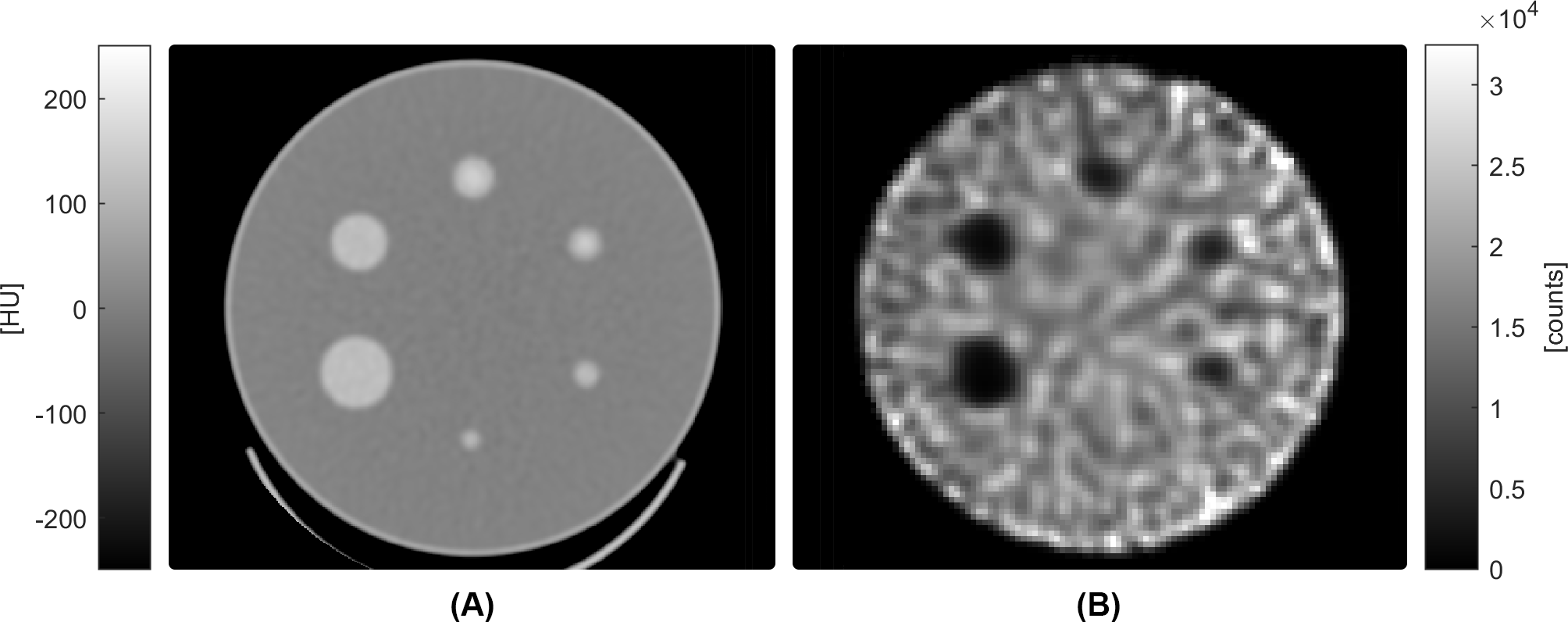

Supplement: Supplementary file 1 — Supplementary Material . [file 40658_2024_671_MOESM1_ESM.docx]
